# Advances in 3D cell culture technologies enabling tissue‐like structures to be created *in vitro*

**DOI:** 10.1111/joa.12257

**Published:** 2014-11-20

**Authors:** Eleanor Knight, Stefan Przyborski

**Affiliations:** ^1^ School of Biological and Biomedical ScienceDurham UniversityDurhamUK

**Keywords:** 3D cell culture, *in vitro*, technology, tissue structure

## Abstract

Research in mammalian cell biology often relies on developing *in vitro* models to enable the growth of cells in the laboratory to investigate a specific biological mechanism or process under different test conditions. The quality of such models and how they represent the behavior of cells in real tissues plays a critical role in the value of the data produced and how it is used. It is particularly important to recognize how the structure of a cell influences its function and how co‐culture models can be used to more closely represent the structure of real tissue. In recent years, technologies have been developed to enhance the way in which researchers can grow cells and more readily create tissue‐like structures. Here we identify the limitations of culturing mammalian cells by conventional methods on two‐dimensional (2D) substrates and review the popular approaches currently available that enable the development of three‐dimensional (3D) tissue models *in vitro*. There are now many ways in which the growth environment for cultured cells can be altered to encourage 3D cell growth. Approaches to 3D culture can be broadly categorized into scaffold‐free or scaffold‐based culture systems, with scaffolds made from either natural or synthetic materials. There is no one particular solution that currently satisfies all requirements and researchers must select the appropriate method in line with their needs. Using such technology in conjunction with other modern resources in cell biology (e.g. human stem cells) will provide new opportunities to create robust human tissue mimetics for use in basic research and drug discovery. Application of such models will contribute to advancing basic research, increasing the predictive accuracy of compounds, and reducing animal usage in biomedical science.

## Introduction

**Figure 1:**
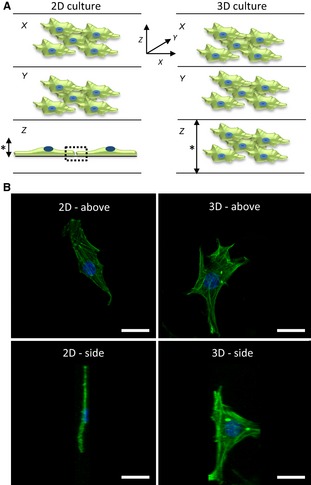
Impact of the physical environment on cell structure. (A) Visualisation of cells for each of the three dimensions (X,Y,Z). In simple terms, X and Y symbolize the length and width of a cell, and Z describes the height. In conventional 2D culture, cells grow as monolayers on a solid substrate; they flatten and possess a low vertical height (left). In contrast, cells cultured in a 3D model maintain a more natural 3D structure and possess more normal dimensions all round (right). Furthermore, the overall height (*) of a conventional 2D monolayer culture is relatively fixed, whereas that of a 3D culture is more versatile, depending on the 3D cell technology used, and can be built up to form multi‐layered tissue‐like structures. Interactions between adjacent cells cultured in 2D are restricted to the periphery of the cells within a single plane (left, dotted box), whereas in 3D models the scope of intercellular contact is all around. (B) Confocal images of a single fibroblast grown in 2D or 3D culture. The cell has been stained with phalloidin to visualize the primary structural elements of the F‐actin cytoskeleton and 4',6‐diamidino‐2‐phenylindole (DAPI) for the nucleus. The images show the shape of a typical cell when visualized from above (top panels) or from the side (bottom panels). Note how thin a cell can become when cultured on a flat substrate as in conventional 2D culture (left) compared with the more normal structure of a cell in a 3D culture model (right). Scale bars: 10 μm. (Images courtesy of Dr. F. Tholozan, Durham University).

It is well established that cells adapt to their surrounding environment by responding to local signals and cues, which in turn has consequences for cell proliferation, differentiation and function (Baker & Chen, 2012). The growth of mammalian cells *in vitro* using traditional culture methods is far removed from the complexities cells encounter in real‐life tissues. One of the major physical differences relates to the shape and geometry cells acquire when grown on a flat substrate such as in a conventional cell culture plate or flask. Growth on two‐dimensional (2D) surfaces results in cell flattening and remodeling of the cell and its internal cytoskeleton (Fig. 1). Such changes have been shown to alter gene expression (Vergani et al. 2004). Cell flattening also affects nuclear shape, which can also lead to differences in gene expression and protein synthesis (Thomas et al. 2002). Accordingly, existing 2D cell culture models are often a poor proxy when used to study cell growth *in vitro* due to their inability to form more natural tissue‐like structures. This has a significant impact on cell performance and consequently influences the results of biological assays. For example, monolayers of cultured cells are thought to be more susceptible to therapeutic agents (Bhadriraju & Chen, 2002; Sun et al. 2006). Furthermore, cell culture on rigid surfaces can enhance cell proliferation but inhibit cell differentiation due to the limited cell interactions (Cukierman et al. 2002). A more appropriately engineered cell culture environment could improve the predictive accuracy of the drug discovery process (Bhadriraju & Chen, 2002) and aid in the understanding of tissue morphogenesis (Yamada & Cukierman, 2007).

Over recent years there has been a gradual development and adoption of technologies that enable cultured cells to acquire or maintain their natural morphology and structure. ‘Three‐dimensional’ (3D) cell culturing has been developed to enhance the structure of cells and physiological relevance of experiments performed *in vitro*. The third dimension allows for greater cell‐to‐cell contact, resulting in increased intercellular signaling, facilitating developmental processes, and allowing cells to differentiate into more complex structures. A 3D environment also enables cells to organize into tissue‐like structures through a more uniform expression of adhesion molecules distributed across the cell surface (Cukierman et al. 2002). In 3D cell culture, receptors and adhesion molecules are more naturally spread more evenly over the cell surface, whereas in 2D culture cells are polarized and binding proteins tend to be concentrated on the ventral surface where they attach to the tissue culture plastic (Bokhari et al. 2007a).

It is important to clearly define what is meant by the term ‘3D cell culture’. 3D cell culture is about providing a suitable micro‐environment for optimal cell growth, differentiation and function, and the ability to create tissue‐like constructs *in vitro*. This is achieved by: (i) allowing individual cells to maintain their normal 3D shape, structure and function with minimal exogenous support and interference; (ii) encouraging cells to form complex interactions with adjacent cells and receive and transmit signals; (iii) enabling a more natural environment for different cell types to foster the creation of native architecture found in tissue structures; and (iv) reducing stress and artificial responses as a result of cell adaptation to flat, 2D growth surfaces.

## Three‐dimensional cell culture technology

**Figure 2:**
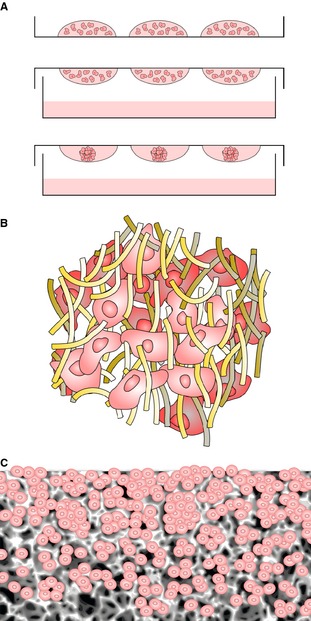
Primary technologies used to culture cells in 3D. (A) Formation of 3D micro‐tissues using hanging drop approach. Droplets of cell suspension are placed on the lid of a Petri dish, which is gently inverted and placed on top of the dish containing media to maintain a humid atmosphere. Suspended cells come together in the apex of the droplet, forming a compact 3D aggregate. (B) 3D culture using hydrogel technology. The cartoon shows cells within a matrix of protein molecules that create a nano‐scale micro‐environment mimicking the structure of the extracellular matrix. Cells are embedded within the proteinous 3D framework within an aqueous‐based gel. (C) Porous solid scaffold supporting 3D cultured cells. Cells enter the porous framework of the solid scaffold where they do not flatten, they maintain their 3D structure, and they bind to one another forming 3D tissue‐like masses.

Various methods have been developed to meet the growing demand for 3D cell culture. There is no panacea or single technology that satisfies the needs of all 3D cell culture and users are required to select the most appropriate model for their cell‐based assay. Approaches to 3D culture can be broadly categorized as scaffold‐free or scaffold‐based culture systems, with scaffolds made from either natural or synthetic materials. For the purposes of this review, we will highlight some of the most popular examples currently used, including aggregate cultures and spheroids, hydrogels, and scaffold‐based technologies (Fig. 2).

### Aggregate cultures and the formation of spheroids

Scaffold‐free systems primarily consist of the formation of multi‐cellular aggregates, often referred to as spheroids, in which cells form their own extracellular matrix components. Such structures can be produced in various ways and using alternative materials. For the purposes of this review we will focus on the formation of spheroids using the hanging drop technique (Keller, 1995), where cells are cultured in a drop of media suspended on the lid of a cell culture dish (Fig. 2A). However, aggregate cultures can also be produced using low adherence substrates. In both cases, cells are unable to adhere to a surface and form clumps in suspension. In hanging drops, the cells form 3D spheroids at the apex of the droplet of media. This approach has multiple applications. It has been shown to permit long‐term cell survival and maintenance of the stem cell phenotype of bone marrow stromal cells (Banerjee & Bhonde, 2006) and it also allows for more homogeneous differentiation compared with standard cell monolayers (De Smedt et al. 2008). It has been proposed that the hanging drop method maintains a high local concentration of endogenous factors such as the hedgehog proteins and is therefore able to sustain tissue function better than monolayer cultures (Szczepny et al. 2009).

The formation of multi‐cellular aggregates is of particular importance in stem cell biology for the *in vitro* differentiation of stem cells. In this case the aggregates are referred to as embryoid bodies (EBs) and can be formed using both the hanging‐drop method and other techniques (Kurosawa, 2007; Antonchuk, 2013). These other techniques allow for the production of uniform‐sized Ebs; this is an important parameter, as EB size has previously been demonstrated to affect cell differentiation (Messana et al. 2008; Bratt‐Leal et al. 2009). Embryonic stem cell‐based aggregates are able to form either simple EBs with morula‐like structures or cystic EBs where a central cavity forms resembling the blastula stage (Abe et al. 1996). The ability to form layered and organized structures that more closely mimic the scale and ordered complexity of real tissues is limited due to problems with long‐term maintenance of EBs. There are also limitations in nutrient and gaseous diffusion and difficulties in media exchange that can lead to necrosis when using the hanging drop method. However, these hypoxic conditions may be advantageous in 3D models used for modeling the development and progression of tumors.

Spheroids are of particular interest to cancer researchers as they contain heterogeneous populations of cells with areas of proliferating cells at the surface of the spheroid and quiescent cells in the center due to limited oxygen and nutrient transport (Mehta et al. 2012). Larger spheroids may contain areas of necrotic cells at the center and may more closely reflect the structure of some tumor types *in vivo* (Yoshii et al. 2011). This can be a useful feature for modeling hypoxia in cancer research (Hirschhaeuser et al. 2010). Aggregate cultures have been used previously to evaluate drug resistance and sensitivity and typically show more resistance to both chemotherapy and radiotherapy when compared with 2D monolayer cultures (Feder‐Mengus et al. 2008). Multicellular spheroids have also been used to successfully culture cancer stem cells (CSCs). These cells are thought to be responsible for the relapse of cancer after treatment (O'Connor et al. 2014). CSCs need to be cultured in 3D to retain specific properties. For example, ovarian cancer spheroids display self‐renewal potential and increased invasiveness compared with cells in monolayers (Wang et al. 2012). Additionally, unlike their 2D counterparts, human colon tumor cells grown in 3D spheroids maintain CD133 expression, expand under serum‐free conditions, initiate xenograft tumors, and display resistance to chemotherapy‐induced apoptosis (Fang et al. 2010).

Suspended 3D spheroids provide a structure that more closely mimics the structural and physiological environment and will allow for the development and testing of therapies that specifically target CSCs. The use of spheroids in the process of cancer research and drug discovery is common. However, the routine use of such models for drug development has been hampered by the lack of standardized procedures to produce uniform spheroids and their incorporation into high‐throughput screening procedures. Recently, several protocols have been developed for the standardization of cancer spheroid formation (Friedrich et al. 2009). Procedures have also been developed that can then be applied to a range of cancer cells and are compatible with existing high throughput systems (Vinci et al. 2012). In addition, multicellular spheroids have been embedded into scaffold‐based technologies of the types highlighted below. One particular study uses collagen hydrogels to embed multicellular aggregates to evaluate both drugs and drug‐device interactions (Charoen et al. 2014).

### Scaffold‐based technologies for 3D cell culture

The use of scaffold‐based 3D culture models extends the range of options available to researchers. 3D scaffolds can be manufactured from a range of natural and synthetic materials. Natural biomaterials are often based on various components of the extracellular matrix (ECM) such as collagen (Baharvand et al. 2006), fibrin (Willerth et al. 2006) and hyaluronic acid (Gerecht et al. 2007) but can also include other naturally derived materials, including silk (Mauney et al. 2007), gelatin (Awad et al. 2004) and alginate (Li et al. 2010). These materials are biocompatible and contain cell adhesion sites. They are also advantageous for tissue engineering applications, as they are biodegradable. For the purposes of cell culture, however, biodegradation of the scaffold may be an unwanted feature since it introduces another variable that is difficult to control and may influence cell activity in unknown ways. Other limitations may include lot‐to‐lot variability and limited mechanical properties.

Scaffolds made from synthetic materials have advantages such as a defined chemical composition and tunable mechanical properties that have been shown to affect cell differentiation (Engler et al. 2006) and cell adhesion (Hayward et al. 2013). Synthetic materials used in 3D scaffolds include biomaterials such as polymers (Gunatillake & Adhikari, 2003) and titanium (van den Dolder et al. 2003), ceramic‐based materials such as bioactive glasses (Lu et al. 2003), and self‐assembled peptides (Garreta et al. 2006). A synthetic scaffold provides reproducibility; it may be inert and non‐degradable or may be designed with tunable degradability that is not possible in naturally derived materials. However, synthetic materials may lack sites for cellular adhesion and may require a coating of ECM proteins to attempt to mimic the niche in which cells reside naturally. There are several different methods for scaffold‐based 3D culture, which can be broadly divided into two approaches – hydrogels and solid scaffolds.

#### Hydrogel technology

**Figure 3:**
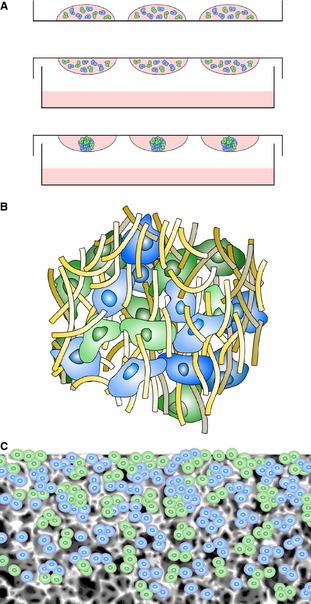
Co‐culture offers an exciting opportunity to create novel 3D tissue‐like models *in vitro*. Each of the three primary technologies developed for 3D culture can be customized for alternative applications containing different cell types. The cartoons show cells of different colors representing alternative cell types within each technology. (A) Cells of different types can be co‐seeded in suspended hanging drops to create micro‐tissue aggregates composed of more than one cell type. The addition of an alternative cell type to a pre‐formed cell aggregate can produce a concentric layered structure. (B) Mixing cells of different types is relatively straightforward using hydrogel technology. Cells can self‐organize and form tissue‐like structures as they migrate within the gel. (C) Similarly, a mixture of different cells can be added directly to the porous scaffold where they self‐arrange within the matrix.

A popular option for 3D culture is the encapsulation of cells in a hydrogel comprising a loose scaffold framework of a cross‐linked natural base material such as agarose, fibrin, collagen or hyaluronic acid with high water content (Tibbitt & Anseth, 2009) (Figs 2B and 3). Hydrogels can be designed to support specific types of cell growth and function by either trapping cells in an artificial ECM protein environment (Heywood et al. 2004; Jongpaiboonkit et al. 2008) or allowing cells to migrate into the interior of the gel from the surface (Topman et al. 2013). This ECM micro‐environment may be modified to incorporate biologically active molecules. Cells may be encapsulated into the gels by self‐assembly, ionic cross‐linking or radical polymerizations by UV exposure. However, hydrogels have inherent disadvantages, such as the use of UV light to cure the gel, which may have a detrimental effect on cells (Nicodemus & Bryant, 2008). In addition, some cells can only be cultured for relatively short periods of time due to problems with the diffusion of nutrients through the hydrogel (Jongpaiboonkit et al. 2008).

##### Common uses of hydrogels in 3D culture

Hydrogels are commonly used in models of branching morphogenesis. This process depends on both chemical and physical properties of the ECM. *In vivo* models are complex, making it difficult to understand the molecular and cellular mechanisms behind the process of tube formation, and 2D *in vitro* models lack the influence of the ECM found within a 3D environment. The advantage of 3D culture for studying the mechanisms of tube formation has been reviewed recently (Zegers, 2014). The formation of branching tubules is commonly studied in either Collagen I matrices (Wells et al. 2013) or reconstituted basement membrane extracts (Debnath et al. 2003) prepared from commercial products such as Matrigel^®^. Comparisons of these two hydrogels have demonstrated that different ECM compositions are required for cell polarization and lumen formation (Santos & Nigam, 1993; Campbell & Watson, 2009). Hydrogels are often used *in vitro* to create models of angiogenesis. Such models have shown potential for the study of both vascular morphogenesis and the preclinical testing of drugs (Zeitlin et al. 2012). Another advantage of hydrogels is their ability to encapsulate and release bioactive agents. Immobilization of regulatory factors in hydrogels has previously been demonstrated to enhance vascular differentiation of human embryonic stem cells (Ferreira et al. 2007). More recently combining hydrogels, bioactive agents and a co‐culture system has provided a model of tumor angiogenesis to further understand the role of endothelial cells in the tumor microenvironment (Chwalek et al. 2014).

Hydrogels have also been used *in vitro* in an attempt to mimic the use of stem cells. Attempts to recreate this niche have involved the use of hyaluronic acid to support the growth of human embryonic stem cells (hESCs) *in vivo* and regulate co‐regulation of gene expression, proliferation and morphogenesis (Toole, 2009). Hyaluronic acid has also been used in hydrogels to maintain the growth of hESCs in culture (Gerecht et al. 2007). This 3D culture environment was used to maintain a state of self‐renewal by hESCs and, with the introduction of angiogenic factors, to induce vascular differentiation. The use of a single component of the ECM in such culture models, however, does not reflect the natural complexity of ECM. Oversimplification of the extracellular micro‐environment can affect cell proliferation, adhesion and phenotype regulation (Cukierman et al. 2002; Hakkinen et al. 2011), which in turn can be detrimental to cellular differentiation. Equally, there are specific ECM proteins that can significantly influence the differentiation of hESCs in 3D models (Battista et al. 2005). While this most likely involves the regulation of signaling via chemical and molecular pathways, there is also evidence that the biomechanical properties of the ECM play an important role (Reilly & Engler, 2010).

An alternative to a simplified single component of ECM is a complex mixture of multiple proteins and associated molecules, such as the well‐known commercial product, Matrigel^®^ (Kleinman & Martin, 2005). Matrigel^®^ is the trade name for a gelatinous protein mixture secreted by Engelbreth‐Holm‐Swarm mouse sarcoma cells and is a form of basement membrane extract. Matrigel^®^ consists of several common ECM proteins including laminin, collagen and entactin, as well as various growth factors. It has previously been used to support a range of cell types in 3D culture (Amatangelo et al. 2013; Lance et al. 2013; Li et al. 2013). Matrigel^®^ remains a popular option for certain cell culture assays but it does have limitations: it is derived from tumor cells; its exact constituents are not clearly defined; it suffers from batch‐to‐batch variation; and it presents handling difficulties when dispensed as a chilled liquid. These issues are disadvantageous for 3D cultures intended for routine predictive drug testing. Moreover, it has been suggested that tumor‐derived ECM mixtures may result in the development of cell adhesion‐mediated drug resistance within the tumor microenvironment (Damiano et al. 1999; Eberle et al. 2011).

One of the primary features of using hydrogels for 3D culture is that cells often grow as isolated aggregates within the gel that in itself is beneficial for certain cell types and assays. For example, tubule formation or modeling the behavior of cancer cells. Hydrogels have also been used to produce well‐stratified 3D tissues using a co‐culture of fibroblast and embryonic stem cell‐derived cells (Hewitt et al. 2009). Organized layered structures composed of alternative cell types can be formed using hydrogel‐based approaches. However, some of the impracticalities concerning the routine use and preparation of hydrogel‐based 3D cultures can restrict this capability.

#### Solid scaffold‐based technology

**Figure 4:**
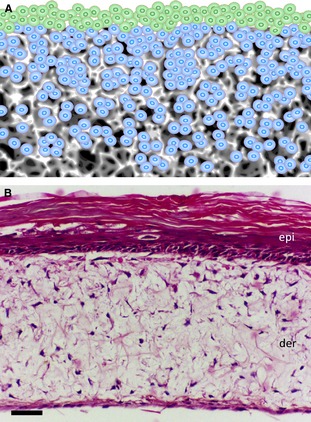
Construction of tissue‐like structures composed of layers of alternative cell types. The presentation of a solid porous scaffold as a membrane provides a suitable platform to construct layers of alternative cell types. Many types of tissue are composed of alternate layers of cells and such tissue architecture can be replicated by seeding cells sequentially onto supporting porous scaffolds. (A) An example of two cell types: those in blue were seeded first and allowed to grow and establish a 3D culture inside the scaffold; those in green were seeded second onto the surface of the scaffold to establish a 3D layer on top. (B) Example of a human skin construct produced by first seeding human primary dermal fibroblasts into a 200‐μm‐thick porous polystyrene scaffold for 7 days, followed by seeding human primary keratinocytes onto the surface and maintaining the culture for a further 21 days at the air–liquid interface. The skin construct matures over time in culture consisting of differentiated epidermal (epi) and supporting dermal (der) components. Scale bar: 50 μm. (Image courtesy of N. Robinson, Durham University).

Seeding cells into a solid scaffold provides a 3D space to support cells, allowing them to create natural 3D tissue‐like structures (Figs 2C and 3). An advantage of solid scaffold‐based technologies is their ability to support 3D culture and produce organized arrangements of cells *in vitro* in a controllable and reproducible manner using methods that are more appropriate for routine use. Cells can readily be seeded into the open pore structure of a pre‐prepared solid scaffold following simple protocols. Commercially available solid scaffolds overcome the impracticalities and variation associated with user‐prepared scaffold materials. Such products are synthetic and free of animal‐based components, supplied sterile and ready to use, and are manufactured according to high quality control procedures, minimizing batch variation, and promoting reproducibility and consistency. Collectively, these features are particularly advantageous where tissue structure is naturally organized, well defined, and has an architecture consisting of discrete layers of alternative cell types. The development of artificial skin constructs is a classic example of the routine application of scaffold technologies to recreate the layered structure of dermal and epidermal components (Fig. 4).

Numerous types of solid scaffold are used for a variety of different applications. For example, a combination of electro‐spinning and other manufacturing techniques has been described for the formation of osteon‐like structures (Chen et al. 2013). Porous scaffold has been shown to support epidermal‐like structures of equine keratinocytes (Sharma et al. 2013), and to produce co‐cultures that can be used to study tumor invasion into the stromal layer (Fischbach et al. 2007). There is an increasing number of such examples in the literature reflecting the rapid growth of these types of technology and their application. With regard to their use for 3D cell culture, such scaffolds can be broadly divided into fibrous or porous matrices manufactured using a range of different techniques and alternative materials.

##### Fibrous scaffolds

A common method for producing fibrous matrices is electro‐spinning. A mesh of fibers is produced by passing a polymer jet through an electric field and collecting the material on a grounded surface (Nisbet et al. 2009). It is possible to use two or more jetting materials either in parallel or in series to produce heterogeneous scaffolds. Biologically active materials can be incorporated into the polymer mesh, for example, the incorporation of DNA into poly(ethylene glycol) electrospun scaffolds (Saraf et al. 2010), the controlled release of antibiotics (Kim et al. 2004) or the delivery anticancer agents (Xie & Wang, 2006). Electrospun scaffolds have been suggested for the support of stem cell cultures, as the 3D arrangement of fibers is thought to mimic the arrangement and scale of collagen fibrils (Lim & Mao, 2009). A unique feature of electrospun scaffolds is the ability to form materials containing aligned fibers. This feature allows cells to adhere and elongate along the fibers, which induces cell alignment and directionality to the cultures (Baker & Mauck, 2007; Wise et al. 2009). In many cases, however, cells grown on fibrous scaffolds are not considered to truly represent 3D cell growth. Cells primarily grow along the fiber and may occasionally bridge the gap between individual fibers. The majority of cells in such cultures are therefore essentially growing around the curvature of a 2D substrate promoted by their adhesions to the rounded fibers (Reilly & Engler, 2010).

##### Porous scaffolds

Porous scaffolds create a 3D microenvironment in which cells enter; they do not flatten out as in conventional 2D cell culture and the cells retain their natural 3D structure. Most important, the cells form contacts and interactions with adjacent cells within the 3D space to create tissue‐like structures. The void and pore dimensions within the material are critical parameters that can influence the ability of cells to fill the 3D space, to bridge gaps, and to fill the voids to create a 3D mass of cells. Scaffolds with a defined void size can be produced using a physical process known as particulate leaching, resulting in a sponge‐like porous polymer (Ma & Choi, 2001). Particulate leaching involves casting polymer around soluble beads known as porogens. Commonly used porogens are salt (Levenberg et al. 2003), sugar (Wei & Ma, 2006) and paraffin spheres (Ma & Choi, 2001). A disadvantage of the particulate leaching process is that it is possible to produce voids that have limited connectivity with adjacent spaces. This lack of inter‐connectivity between voids can lead to a heterogeneous culture of isolated cells within the 3D environment of the porous scaffold. Levenberg et al. (2003) used salt‐leached poly(lactic‐co‐glycolic acid) (PLGA) and poly(l‐lactic acid) (PLA) to form porous scaffolds to create an artificial 3D microenvironment for differentiating human embryonic stem cells. Although this was successful in part, they also demonstrated the difficulties of getting the cells to infiltrate throughout the scaffold (Levenberg et al. 2003). An alternative strategy to producing porous scaffolds involves emulsion templating (Bokhari et al. 2007c). As with particulate leaching, it is possible to produce pores of defined size but more finely control the properties of the material (Carnachan et al. 2006). Unlike porogen leaching, all voids are connected to adjacent voids via interconnecting pores, resulting in a highly porous material which cells can populate in a homogeneous manner creating substantial 3D cultures.

##### Use of different materials for the production of solid scaffolds

Various materials have been used to create different types of scaffold that have subsequently been developed into a range of formats and presentations. Natural substrates such as seaweed‐derived alginate have been used to support 3D growth either by encapsulation (Zimmermann et al. 2007) or fabrication into a macro‐porous scaffold (Dvir‐Ginzberg et al. 2008). This method allows the growth of cells as individual spherical masses within large voids of 50–100 μm. However, such growth is not homogeneous throughout the material and the large thickness of the scaffold is not supportive of efficient mass transfer of oxygen and nutrients. Biodegradable materials such as poly(lactic acid) and poly(glycolic acid) and their co‐polymer [poly(lactic‐*co*‐glycolic acid)] have been developed previously for use in 3D cell growth (Mikos et al. 1993). These materials were originally developed for use in tissue engineering applications, as their ability to degrade, aids the integration of transplanted cells within host tissues. Although this is an advantage for tissue repair, biodegradability can be detrimental for *in vitro* studies. Scaffold degradation can lead to the release of by‐products such as lactic acid, and localized areas of high concentration of by‐products can subsequently influence cell behavior. This is especially important during stem cell culture, as the build‐up of waste products can lead to a decrease in levels of pluripotency markers and the onset of differentiation (Ouyang et al. 2007). Moreover, biodegradable materials are not practical for routine 3D cell culture where issues such as shelf life, storage and product consistency need to be taken into consideration.

The use of inert non‐degradable materials overcomes these problems and they can be engineered into highly porous scaffolds suitable for routine 3D cell culture. Such scaffolds can be manufactured using the techniques previously highlighted such as electrospinning (Sun et al. 2007), particulate leaching (Aydin et al. 2009) and emulsion templating (Carnachan et al. 2006; Bokhari et al. 2007c), but can also be formed using alternative methods including gas foaming technology (Salerno et al. 2009). These scaffolds provide a large internal volume and 3D space that cells can occupy and form tissue‐like structures. Variations in scaffold manufacturing technique have led to small‐scale advances in materials development and application to cell biology but very few have been developed into a commercially successful process to consistently manufacture scaffolds for biological applications *in vitro* and routine 3D cell culture. Development of such a consumable product is an essential step if 3D culture is to be accepted by the scientific community and commonly used in the cell culture laboratory. The exception is emulsion templating, a long established technology for producing highly porous polymeric scaffolds. Emulsion templating has subsequently been optimized to produce highly porous scaffolds made from cross‐linked polystyrene which has been sectioned into 200‐μm‐thick membranes for use in routine 3D cell culture (Maltman & Przyborski, 2010; Knight et al. 2011). Such materials have been used to culture a range of cell types in 3D, including hepatocytes (Bokhari et al. 2007a,b; Schutte et al. 2011), osteoblasts (Bokhari et al. 2007c) and pluripotent stem cell‐derived neurons (Hayman et al. 2004, 2005). The 3D culture environment provided by this type of scaffold has demonstrated increased functionality compared with conventional 2D cultures (Burkard et al. 2012) and cytotoxic agents have displayed lower apparent cytotoxicity (Alayoubi et al. 2013).

Polystyrene is an attractive substrate for 3D cell culture since it is familiar to the user, is inert, and does not degrade during normal use. This makes the scaffold useful for *in vitro* testing when a consistent cell culture environment is required. However, polystyrene lacks biochemical stimuli (e.g. cell anchorage‐dependent molecules), although this can partly be addressed by coating such scaffolds with known extracellular matrix proteins. Polystyrene is also stiff and lacks the biomechanical properties found in soft tissues. In this instance a hydrogel‐based technology may be a more appropriate choice where the stiffness of the scaffold can be controlled. A balance therefore needs to be struck between the needs of the model and the convenience of its use.

Solid porous scaffolds are particularly useful for the co‐culture of different cell types in layered arrangements in close proximity (Fig. 4). There are many examples of tissues in the body where cells of alternative types are arranged as adjacent layers. Such tissue architecture is common in epithelia and certain organ systems. Modern 3D cell culture methods can now enable the recreation of these structures, resulting in realistic tissue constructs *in vitro*. An example of a 3D human skin construct is shown in Fig. 4 where dermal fibroblasts are seeded within an inert scaffold supporting a layer of stratified keratinocytes at the air–liquid interface on the surface. The human dermal fibroblasts produce extracellular matrix proteins within the scaffold including significant amounts of endogenous collagen, thus removing the need for an exogenous collagen gel.

## Summary

Advances in technology have led to new opportunities for growing cells in culture and the creation of 3D tissue‐like constructs. This is primarily a consequence of inter‐disciplinary research between cell biology and the biophysical sciences, introducing new materials and methods of manufacture to create platforms tailored to support 3D cell growth *in vitro*. The culture of cells in 3D is advancing rapidly, as reflected in the growing numbers of the publications in the scientific literature. The success of this technology will depend on the adoption, validation and application of these new approaches. This is likely to take time as the scientific community recognizes the limitations of conventional 2D cell culture and appreciates the value of new ways to reliably culture cells in 3D. The creation of tissue‐like constructs in a reliable and reproducible manner according to clearly defined protocols is essential to encourage users to adopt these new approaches. A potential issue is a reliable source of cells for such models, particularly human cells. Stem cell science offers an exciting opportunity whereby a renewable source of human material can be differentiated into cells of interest. However, differentiation of tissue is complex and occurs naturally within a 3D context. The ability to combine stem cell research and 3D culture technology therefore opens exciting new prospects for the creation of human tissue *in vitro* for use in basic research, drug discovery and safety screening. Inroads in this area have already been made, showing how a combination of stem cells and scaffolds is beneficial for the construction of tissues *in vivo* and for cell delivery (Willerth & Sakiyama‐Elbert, 2008). The opportunities to recreate tissue structure *in vitro* using human stem cell‐derived materials will have many advantages, advancing research, increasing the efficiency of drug discovery, and reducing the reliance on animal models. This field has enormous potential and there will be many developments over the coming years that will radically influence how cells are cultured in the laboratory.

## Conflict of interest

Professor Przyborski holds a Chair in Cell Technology at Durham University. He is also the founder of Reinnervate Limited, a business involved with the development of 3D cell culture technology. He is no longer a shareholder or legal director of this company.

## References

[joa12257-bib-0001] Abe K , Niwa H , Iwase K , et al. (1996) Endoderm‐specific gene expression in embryonic stem cells differentiated to embryoid bodies. Exp Cell Res 229, 27–34. 8940246 10.1006/excr.1996.0340

[joa12257-bib-0002] Alayoubi A , Alqahtani S , Kaddoumi A , et al. (2013) Effect of PEG surface conformation on anticancer activity and blood circulation of nanoemulsions loaded with tocotrienol‐rich fraction of palm oil. AAPS J 15, 1168–1179. 23990503 10.1208/s12248-013-9525-z PMC3787212

[joa12257-bib-0003] Amatangelo MD , Garipov A , Li H , et al. (2013) Three‐dimensional culture sensitizes epithelial ovarian cancer cells to EZH2 methyltransferase inhibition. Cell Cycle 12, 2113–2119. 23759589 10.4161/cc.25163 PMC3737313

[joa12257-bib-0004] Antonchuk J (2013) Formation of embryoid bodies from human pluripotent stem cells using AggreWell plates. Methods Mol Biol, 946, 523–533. 23179853 10.1007/978-1-62703-128-8_32

[joa12257-bib-0005] Awad HA , Wickham MQ , Leddy HA , et al. (2004) Chondrogenic differentiation of adipose‐derived adult stem cells in agarose, alginate, and gelatin scaffolds. Biomaterials 25, 3211–3222. 14980416 10.1016/j.biomaterials.2003.10.045

[joa12257-bib-0006] Aydin HM , El Haj AJ , Piskin E , et al. (2009) Improving pore interconnectivity in polymeric scaffolds for tissue engineering. J Tissue Eng Regen Med 3, 470–476. 19530258 10.1002/term.187

[joa12257-bib-0007] Baharvand H , Hashemi SM , Ashtian SK , et al. (2006) Differentiation of human embryonic stem cells into hepatocytes in 2D and 3D culture systems in vitro. Int J Dev Biol 50, 645–652. 16892178 10.1387/ijdb.052072hb

[joa12257-bib-0008] Baker BM , Chen CS (2012) Deconstructing the third dimension – how 3D culture microenvironments alter cellular cues. J Cell Sci 125, 3015–3024. 22797912 10.1242/jcs.079509 PMC3434846

[joa12257-bib-0009] Baker BM , Mauck RL (2007) The effect of nanofiber alignment on the maturation of engineered meniscus constructs. Biomaterials 28, 1967–1977. 17250888 10.1016/j.biomaterials.2007.01.004 PMC1847368

[joa12257-bib-0010] Banerjee M , Bhonde RR (2006) Application of hanging drop technique for stem cell differentiation and cytotoxicity studies. Cytotechnology 51, 1–5. 19002889 10.1007/s10616-006-9001-z PMC3449481

[joa12257-bib-0011] Battista S , Guarnieri D , Borselli C , et al. (2005) The effect of matrix composition of 3D constructs on embryonic stem cell differentiation. Biomaterials 26, 6194–6207. 15921736 10.1016/j.biomaterials.2005.04.003

[joa12257-bib-0012] Bhadriraju K , Chen CS (2002) Engineering cellular microenvironments to cell‐based drug testing improve cell‐based drug testing. Drug Discov Today 7, 612–620. 12047872 10.1016/s1359-6446(02)02273-0

[joa12257-bib-0013] Bokhari M , Carnachan RJ , Cameron NR , et al. (2007a) Culture of HepG2 liver cells on three dimensional polystyrene scaffolds enhances cell structure and function during toxicological challenge. J Anat 211, 567–576. 17711423 10.1111/j.1469-7580.2007.00778.x PMC2375833

[joa12257-bib-0014] Bokhari M , Carnachan RJ , Cameron NR , et al. (2007b) Novel cell culture device enabling three‐dimensional cell growth and improved cell function. Biochem Biophys Res Commun 354, 1095–1100. 17276400 10.1016/j.bbrc.2007.01.105

[joa12257-bib-0015] Bokhari M , Carnachan RJ , Przyborski SA , et al. (2007c) Emulsion‐templated porous polymers as scaffolds for three dimensional cell culture: effect of synthesis parameters on scaffold formation and homogeneity. J Mater Chem 17, 4088–4094.

[joa12257-bib-0016] Bratt‐Leal AM , Carpenedo RL , McDevitt TC (2009) Engineering the embryoid body microenvironment to direct embryonic stem cell differentiation. Biotechnol Prog 25, 43–51. 19198003 10.1002/btpr.139 PMC2693014

[joa12257-bib-0017] Burkard A , Dahn C , Heinz S , et al. (2012) Generation of proliferating human hepatocytes using upcyte (R) technology: characterisation and applications in induction and cytotoxicity assays. Xenobiotica 42, 939–956. 22524704 10.3109/00498254.2012.675093

[joa12257-bib-0019] Campbell JJ , Watson CJ (2009) Three‐dimensional culture models of mammary gland. Organogenesis 5, 43–49. 19794898 10.4161/org.5.2.8321 PMC2710524

[joa12257-bib-0020] Carnachan RJ , Bokhari M , Przyborski SA , et al. (2006) Tailoring the morphology of emulsion‐templated porous polymers. Soft Matter 2, 608–616. 10.1039/b603211g 32680240

[joa12257-bib-0021] Charoen KM , Fallica B , Colson YL , et al. (2014) Embedded multicellular spheroids as a biomimetic 3D cancer model for evaluating drug and drug‐device combinations. Biomaterials 35, 2264–2271. 24360576 10.1016/j.biomaterials.2013.11.038 PMC3923358

[joa12257-bib-0022] Chen XN , Ergun A , Gevgilili H , et al. (2013) Shell‐core bi‐layered scaffolds for engineering of vascularized osteon‐like structures. Biomaterials 34, 8203–8212. 23896002 10.1016/j.biomaterials.2013.07.035

[joa12257-bib-0023] Chwalek K , Tsurkan MV , Freudenberg U , et al. (2014) Glycosaminoglycan‐based hydrogels to modulate heterocellular communication in *in vitro* angiogenesis models. Sci Rep 4, 4414. doi: 10.1038/srep04414. 24643064 10.1038/srep04414 PMC3958722

[joa12257-bib-0024] Cukierman E , Pankov R , Yamada KM (2002) Cell interactions with three‐dimensional matrices. Curr Opin Cell Biol 14, 633–639. 12231360 10.1016/s0955-0674(02)00364-2

[joa12257-bib-0025] Damiano JS , Cress AE , Hazlehurst LA , et al. (1999) Cell adhesion mediated drug resistance (CAM‐DR): role of integrins and resistance to apoptosis in human myeloma cell lines. Blood 93, 1658–1667. 10029595 PMC5550098

[joa12257-bib-0026] De Smedt A , Steemans M , De Boeck M , et al. (2008) Optimisation of the cell cultivation methods in the embryonic stem cell test results in an increased differentiation potential of the cells into strong beating myocard cells. Toxicol In Vitro 22, 1789–1796. 18672049 10.1016/j.tiv.2008.07.003

[joa12257-bib-0027] Debnath J , Muthuswamy SK , Brugge JS (2003) Morphogenesis and oncogenesis of MCF‐10A mammary epithelial acini grown in three‐dimensional basement membrane cultures. Methods 30, 256–268. 12798140 10.1016/s1046-2023(03)00032-x

[joa12257-bib-0028] van den Dolder J , Spauwen PHM , Jansen JA (2003) Evaluation of various seeding techniques for culturing osteogenic cells on titanium fiber mesh. Tissue Eng 9, 315–325. 12740094 10.1089/107632703764664783

[joa12257-bib-0029] Dvir‐Ginzberg M , Elkayam T , Cohen S (2008) Induced differentiation and maturation of newborn liver cells into functional hepatic tissue in macroporous alginate scaffolds. FASEB J 22, 1440–1449. 18070820 10.1096/fj.07-9277com

[joa12257-bib-0030] Eberle KE , Sansing HA , Szaniszlo P , et al. (2011) Carcinoma matrix controls resistance to cisplatin through talin regulation of NF‐kB. PLoS ONE 6, e21496. doi: 10.1371/journal.pone.0021496. [Epub 2011 Jun 24]. 21720550 10.1371/journal.pone.0021496 PMC3123362

[joa12257-bib-0031] Engler AJ , Sen S , Sweeney HL , et al. (2006) Matrix elasticity directs stem cell lineage specification. Cell 126, 677–689. 16923388 10.1016/j.cell.2006.06.044

[joa12257-bib-0032] Fang DD , Kim YJ , Lee CN , et al. (2010) Expansion of CD133^+^ colon cancer cultures retaining stem cell properties to enable cancer stem cell target discovery. Br J Cancer 102, 1265–1275. 20332776 10.1038/sj.bjc.6605610 PMC2855999

[joa12257-bib-0033] Feder‐Mengus C , Ghosh S , Reschner A , et al. (2008) New dimensions in tumor immunology: what does 3D culture reveal? Trends Mol Med 14, 333–340. 18614399 10.1016/j.molmed.2008.06.001

[joa12257-bib-0034] Ferreira LS , Gerecht S , Fuller J , et al. (2007) Bioactive hydrogel scaffolds for controllable vascular differentiation of human embryonic stem cells. Biomaterials 28, 2706–2717. 17346788 10.1016/j.biomaterials.2007.01.021 PMC1903348

[joa12257-bib-0035] Fischbach C , Chen R , Matsumoto T , et al. (2007) Engineering tumors with 3D scaffolds. Nat Methods 4, 855–860. 17767164 10.1038/nmeth1085

[joa12257-bib-0036] Friedrich J , Seidel C , Ebner R , et al. (2009) Spheroid‐based drug screen: considerations and practical approach. Nat Protoc 4, 309–324. 19214182 10.1038/nprot.2008.226

[joa12257-bib-0037] Garreta E , Genove E , Borros S , et al. (2006) Osteogenic differentiation of mouse embryonic stem cells and mouse embryonic fibroblasts in a three‐dimensional self‐assembling peptide scaffold. Tissue Eng 12, 2215–2227. 16968162 10.1089/ten.2006.12.2215

[joa12257-bib-0038] Gerecht S , Burdick JA , Ferreira LS , et al. (2007) Hyaluronic acid hydrogel for controlled self‐renewal and differentiation of human embryonic stem cells. Proc Natl Acad Sci U S A 104, 11298–11303. 17581871 10.1073/pnas.0703723104 PMC2040893

[joa12257-bib-0039] Gunatillake PA , Adhikari R (2003) Biodegradable synthetic polymers for tissue engineering. Eur Cell Mater 5, 1–16. 14562275 10.22203/ecm.v005a01

[joa12257-bib-0040] Hakkinen KM , Harunaga JS , Doyle AD , et al. (2011) Direct comparisons of the morphology, migration, cell adhesions, and actin cytoskeleton of fibroblasts in four different three‐dimensional extracellular matrices. Tissue Eng Part A 17, 713–724. 20929283 10.1089/ten.tea.2010.0273 PMC3043991

[joa12257-bib-0041] Hayman MW , Smith KH , Cameron NR , et al. (2004) Enhanced neurite outgrowth by human neurons grown on solid three‐dimensional scaffolds. Biochem Biophys Res Commun 314, 483–488. 14733931 10.1016/j.bbrc.2003.12.135

[joa12257-bib-0042] Hayman MW , Smith KH , Cameron NR , et al. (2005) Growth of human stem cell‐derived neurons on solid three‐dimensional polymers. J Biochem Biophys Methods 62, 231–240. 15733583 10.1016/j.jbbm.2004.12.001

[joa12257-bib-0043] Hayward AS , Sano N , Przyborski SA , et al. (2013) Acrylic‐acid‐functionalized PolyHIPE scaffolds for use in 3D cell culture. Macromol Rapid Commun 34, 1844–1849. 24243821 10.1002/marc.201300709

[joa12257-bib-0044] Hewitt KJ , Shamis Y , Carlson MW , et al. (2009) Three‐dimensional epithelial tissues generated from human embryonic stem cells. Tissue Eng Part A 15, 3417–3426. 19405784 10.1089/ten.tea.2009.0060 PMC2792058

[joa12257-bib-0045] Heywood HK , Sembi PK , Lee DA , et al. (2004) Cellular utilization determines viability and matrix distribution profiles in chondrocyte‐seeded alginate constructs. Tissue Eng 10, 1467–1479. 15588406 10.1089/ten.2004.10.1467

[joa12257-bib-0046] Hirschhaeuser F , Menne H , Dittfeld C , et al. (2010) Multicellular tumor spheroids: an underestimated tool is catching up again. J Biotechnol, 148, 3–15. 20097238 10.1016/j.jbiotec.2010.01.012

[joa12257-bib-0047] Jongpaiboonkit L , King WJ , Lyons GE , et al. (2008) An adaptable hydrogel array format for 3‐dimensional cell culture and analysis. Biomaterials 29, 3346–3356. 18486205 10.1016/j.biomaterials.2008.04.040 PMC2490817

[joa12257-bib-0048] Keller GM (1995) In‐vitro differentiation of embryonic stem‐cells. Curr Opin Cell Biol 7, 862–869. 8608017 10.1016/0955-0674(95)80071-9

[joa12257-bib-0049] Kim K , Luu YK , Chang C , et al. (2004) Incorporation and controlled release of a hydrophilic antibiotic using poly(lactide‐co‐glycolide)‐based electrospun nanofibrous scaffolds. J Control Release 98, 47–56. 15245888 10.1016/j.jconrel.2004.04.009

[joa12257-bib-0050] Kleinman HK , Martin GR (2005) Matrigel: basement membrane matrix with biological activity. Semin Cancer Biol 15, 378–386. 15975825 10.1016/j.semcancer.2005.05.004

[joa12257-bib-0051] Knight E , Murray B , Carnachan R , et al. (2011) Alvetex: polystyrene scaffold technology for routine three dimensional cell culture In: 3D Cell Culture: Methods and Protocols. (ed. Haycock JW), pp. 323–340, Totowa: Humana Press Inc. 10.1007/978-1-60761-984-0_20 21042981

[joa12257-bib-0052] Kurosawa H (2007) Methods for inducing embryoid body formation: in vitro differentiation system of embryonic stem cells. J Biosci Bioeng 103, 389–398. 17609152 10.1263/jbb.103.389

[joa12257-bib-0053] Lance A , Yang CC , Swamydas M , et al. (2013) Increased extracellular matrix density decreases MCF10A breast cell acinus formation in 3D culture conditions. J Tissue Eng Regen Med. doi: 10.1002/term.1675. [Epub ahead of print] 10.1002/term.1675 23404906

[joa12257-bib-0054] Levenberg S , Huang NF , Lavik E , et al. (2003) Differentiation of human embryonic stem cells on three‐dimensional polymer scaffolds. Proc Natl Acad Sci U S A 100, 12741–12746. 14561891 10.1073/pnas.1735463100 PMC240688

[joa12257-bib-0055] Li ZS , Leung M , Hopper R , et al. (2010) Feeder‐free self‐renewal of human embryonic stem cells in 3D porous natural polymer scaffolds. Biomaterials 31, 404–412. 19819007 10.1016/j.biomaterials.2009.09.070

[joa12257-bib-0056] Li HH , Chen L , Zhang MJ , et al. (2013) Three‐dimensional culture and identification of human eccrine sweat glands in matrigel basement membrane matrix. Cell Tissue Res 354, 897–902. 23996202 10.1007/s00441-013-1718-3

[joa12257-bib-0057] Lim SH , Mao HQ (2009) Electrospun scaffolds for stem cell engineering. Adv Drug Deliv Rev 61, 1084–1096. 19647024 10.1016/j.addr.2009.07.011

[joa12257-bib-0058] Lu HH , El‐Amin SF , Scott KD , et al. (2003) Three‐dimensional, bioactive, biodegradable, polymer‐bioactive glass composite scaffolds with improved mechanical properties support collagen synthesis and mineralization of human osteoblast‐like cells in vitro. J Biomed Mater Res A 64A, 465–474. 10.1002/jbm.a.10399 12579560

[joa12257-bib-0059] Ma PX , Choi JW (2001) Biodegradable polymer scaffolds with well‐defined interconnected spherical pore network. Tissue Eng 7, 23–33. 11224921 10.1089/107632701300003269

[joa12257-bib-0060] Maltman DJ , Przyborski SA (2010) Developments in three‐dimensional cell culture technology aimed at improving the accuracy of in vitro analyses. Biochem Soc Trans 38, 1072–1075. 20659006 10.1042/BST0381072

[joa12257-bib-0061] Mauney JR , Nguyen T , Gillen K , et al. (2007) Engineering adipose‐like tissue in vitro and in vivo utilizing human bone marrow and adipose‐derived mesenchymal stem cells with silk fibroin 3D scaffolds. Biomaterials 28, 5280–5290. 17765303 10.1016/j.biomaterials.2007.08.017 PMC2695965

[joa12257-bib-0062] Mehta G , Hsiao AY , Ingram M , et al. (2012) Opportunities and challenges for use of tumor spheroids as models to test drug delivery and efficacy. J Control Release 164, 192–204. 22613880 10.1016/j.jconrel.2012.04.045 PMC3436947

[joa12257-bib-0063] Messana JM , Hwang NS , Coburn J , et al. (2008) Size of the embryoid body influences chondrogenesis of mouse embryonic stem cells. J Tissue Eng Regen Med 2, 499–506. 18956411 10.1002/term.125

[joa12257-bib-0064] Mikos AG , Sarakinos G , Leite SM , et al. (1993) Laminated 3‐dimensional biodegradable foams for use in tissue engineering. Biomaterials 14, 323–330. 8507774 10.1016/0142-9612(93)90049-8

[joa12257-bib-0065] Nicodemus GD , Bryant SJ (2008) Cell encapsulation in biodegradable hydrogels for tissue engineering applications. Tissue Eng Part B Rev 14, 149–165. 18498217 10.1089/ten.teb.2007.0332 PMC2962861

[joa12257-bib-0066] Nisbet DR , Forsythe JS , Shen W , et al. (2009) Review paper: a review of the cellular response on electrospun nanofibers for tissue engineering. J Biomater Appl 24, 7–29. 19074469 10.1177/0885328208099086

[joa12257-bib-0067] O'Connor ML , Xiang D , Shigdar S , et al. (2014) Cancer stem cells: a contentious hypothesis now moving forward. Cancer Lett 344, 180–187. 24333726 10.1016/j.canlet.2013.11.012

[joa12257-bib-0068] Ouyang A , Ng R , Yang ST (2007) Long‐term culturing of undifferentiated embryonic stem cells in conditioned media and three‐dimensional fibrous matrices without extracellular matrix coating. Stem Cells 25, 447–454. 17023515 10.1634/stemcells.2006-0322

[joa12257-bib-0069] Reilly GC , Engler AJ (2010) Intrinsic extracellular matrix properties regulate stem cell differentiation. J Biomech 43, 55–62. 19800626 10.1016/j.jbiomech.2009.09.009

[joa12257-bib-0070] Salerno A , Oliviero M , Di Maio E , et al. (2009) Design of porous polymeric scaffolds by gas foaming of heterogeneous blends. J Mater Sci Mater Med 20, 2043–2051. 19430895 10.1007/s10856-009-3767-4

[joa12257-bib-0071] Santos OFP , Nigam SK (1993) HGF‐induced tubulogenesis and branching of epithelial cells is modulated by extracellular matrix and TGF‐β. Dev Biol 160, 293–302. 8253265 10.1006/dbio.1993.1308

[joa12257-bib-0072] Saraf A , Baggett LS , Raphael RM , et al. (2010) Regulated non‐viral gene delivery from coaxial electrospun fiber mesh scaffolds. J Control Release 143, 95–103. 20006660 10.1016/j.jconrel.2009.12.009 PMC2840180

[joa12257-bib-0073] Schutte M , Fox B , Baradez MO , et al. (2011) Rat primary hepatocytes show enhanced performance and sensitivity to acetaminophen during three‐dimensional culture on a polystyrene scaffold designed for routine use. Assay Drug Dev Technol 9, 475–486. 21675871 10.1089/adt.2011.0371

[joa12257-bib-0074] Sharma R , Barakzai SZ , Taylor SE , et al. (2013) Epidermal‐like architecture obtained from equine keratinocytes in three‐dimensional cultures. J Tissue Eng Regen Med. doi: 10.1002/term.1788. [Epub ahead of print] 10.1002/term.1788 23897780

[joa12257-bib-0075] Sun T , Jackson S , Haycock JW , et al. (2006) Culture of skin cells in 3D rather than 2D improves their ability to survive exposure to cytotoxic agents. J Biotechnol 122, 372–381. 16446003 10.1016/j.jbiotec.2005.12.021

[joa12257-bib-0076] Sun T , Norton D , McKean RJ , et al. (2007) Development of a 3D cell culture system for investigating cell interactions with electrospun fibers. Biotechnol Bioeng 97, 1318–1328. 17171721 10.1002/bit.21309

[joa12257-bib-0077] Szczepny A , Hogarth CA , Young J , et al. (2009) Identification of hedgehog signaling outcomes in mouse testis development using a hanging drop‐culture system. Biol Reprod 80, 258–263. 18843087 10.1095/biolreprod.108.067926 PMC2848731

[joa12257-bib-0078] Thomas CH , Collier JH , Sfeir CS , et al. (2002) Engineering gene expression and protein synthesis by modulation of nuclear shape. Proc Natl Acad Sci U S A 99, 1972–1977. 11842191 10.1073/pnas.032668799 PMC122304

[joa12257-bib-0079] Tibbitt MW , Anseth KS (2009) Hydrogels as extracellular matrix mimics for 3D cell culture. Biotechnol Bioeng 103, 655–663. 19472329 10.1002/bit.22361 PMC2997742

[joa12257-bib-0080] Toole BP (2009) Hyaluronan: from extracellular glue to cell signaling cue. Int J Exp Pathol 90, A96.

[joa12257-bib-0081] Topman G , Shoham N , Sharabani‐Yosef O , et al. (2013) A new technique for studying directional cell migration in a hydrogel‐based three‐dimensional matrix for tissue engineering model systems. Micron, 51, 9–12. 23896652 10.1016/j.micron.2013.06.002

[joa12257-bib-0082] Vergani L , Grattarola M , Nicolini C (2004) Modifications of chromatin structure and gene expression following induced alterations of cellular shape. Int J Biochem Cell Biol 36, 1447–1461. 15147724 10.1016/j.biocel.2003.11.015

[joa12257-bib-0083] Vinci M , Gowan S , Boxall F , et al. (2012) Advances in establishment and analysis of three‐dimensional tumor spheroid‐based functional assays for target validation and drug evaluation. BMC Biol 10, 29. doi: 10.1186/1741‐7007‐10‐29. 22439642 10.1186/1741-7007-10-29 PMC3349530

[joa12257-bib-0084] Wang LJ , Mezencev R , Bowen NJ , et al. (2012) Isolation and characterization of stem‐like cells from a human ovarian cancer cell line. Mol Cell Biochem 363, 257–268. 22160925 10.1007/s11010-011-1178-6

[joa12257-bib-0085] Wei G , Ma PX (2006) Macroporous and nanofibrous polymer scaffolds and polymer/bone‐like apatite composite scaffolds generated by sugar spheres. J Biomed Mater Res A 78A, 306–315. 10.1002/jbm.a.30704 16637043

[joa12257-bib-0086] Wells EK , Yarborough O , Lifton RP , et al. (2013) Epithelial morphogenesis of MDCK cells in three‐dimensional collagen culture is modulated by interleukin‐8. Am J Physiol Cell Physiol 304, C966–C975. 23485708 10.1152/ajpcell.00261.2012 PMC3651639

[joa12257-bib-0087] Willerth SM , Sakiyama‐Elbert SE (2008) Combining stem cells and biomaterial scaffolds for constructing tissues and cell delivery. 20614610

[joa12257-bib-0088] Willerth SM , Arendas KJ , Gottlieb DI , et al. (2006) Optimization of fibrin scaffolds for differentiation of murine embryonic stem cells into neural lineage cells. Biomaterials 27, 5990–6003. 16919326 10.1016/j.biomaterials.2006.07.036 PMC1794024

[joa12257-bib-0089] Wise JK , Yarin AL , Megaridis CM , et al. (2009) Chondrogenic differentiation of human mesenchymal stem cells on oriented nanofibrous scaffolds: engineering the superficial zone of articular cartilage. Tissue Eng Part A 15, 913–921. 18767972 10.1089/ten.tea.2008.0109 PMC2810270

[joa12257-bib-0090] Xie JW , Wang CH (2006) Electrospun micro‐ and nanofibers for sustained delivery of paclitaxel to treat C6 glioma in vitro. Pharm Res 23, 1817–1826. 16841195 10.1007/s11095-006-9036-z

[joa12257-bib-0091] Yamada KM , Cukierman E (2007) Modeling tissue morphogenesis and cancer in 3D. Cell 130, 601–610. 17719539 10.1016/j.cell.2007.08.006

[joa12257-bib-0092] Yoshii Y , Waki A , Yoshida K , et al. (2011) The use of nanoimprinted scaffolds as 3D culture models to facilitate spontaneous tumor cell migration and well‐regulated spheroid formation. Biomaterials 32, 6052–6058. 21640378 10.1016/j.biomaterials.2011.04.076

[joa12257-bib-0093] Zegers MM (2014) 3D in vitro cell culture models of tube formation. Semin Cell Dev Biol 31, 132–140. http://dx.doi.org/10.1016/j.semcdb.2014.02.016 24613912 10.1016/j.semcdb.2014.02.016

[joa12257-bib-0094] Zeitlin BD , Dong ZH , Nor JE (2012) RAIN‐Droplet: a novel 3D in vitro angiogenesis model. Lab Invest 92, 988–998. 22565576 10.1038/labinvest.2012.77 PMC4043634

[joa12257-bib-0095] Zimmermann H , Shirley SG , Zimmermann U (2007) Alginate‐based encapsulation of cells: past, present, and future. Curr DiabRep 7, 314–320. 10.1007/s11892-007-0051-1 17686410

